# Author Correction: Dynamic ubiquitylation of Sox2 regulates proteostasis and governs neural progenitor cell differentiation

**DOI:** 10.1038/s41467-018-07984-3

**Published:** 2019-01-08

**Authors:** Chun-Ping Cui, Yuan Zhang, Chanjuan Wang, Fang Yuan, Hongchang Li, Yuying Yao, Yuhan Chen, Chunnan Li, Wenyi Wei, Cui Hua Liu, Fuchu He, Yan Liu, Lingqiang Zhang

**Affiliations:** 1 State Key Laboratory of Proteomics, National Center of Protein Sciences (Beijing), Beijing Institute of Lifeomics, 100850 Beijing, China; 2 State Key Laboratory of Reproductive Medicine, Institute for Stem Cell and Neural Regeneration, School of Pharmacy, Nanjing Medical University, Nanjing, China; 3 Institute for Stem Cell and Regeneration, Chinese Academy of Science, 100101 Beijing, China; 4 Department of Pathology, Beth Israel Deaconess Medical Center, Harvard Medical School, Boston, USA; 5 CAS Key Laboratory of Pathogenic Microbiology and Immunology, Institute of Microbiology, Chinese Academy of Sciences, 100101 Beijing, China

Correction to: *Nature Communications*; 10.1038/s41467-018-07025-z; published online 7 November 2018

The original version of this Article contained an error in Fig. 7. In panel c, the lanes of the western blots were incorrectly labelled ‘+/+ +/+ −/− −/− +/+ +/+ −/− −/−’. This has been corrected in both the PDF and HTML versions of the Article.

