# Supplementary material for: Dynamic ubiquitylation of Sox2 regulates proteostasis and governs neural progenitor cell differentiation
Source: Nat Commun. 2018 Nov 7;9:4648. doi: 10.1038/s41467-018-07025-z (PMC6220269; doi:10.1038/s41467-018-07025-z)
Supplement: Supplementary file 1 — Supplementary Information [file 41467_2018_7025_MOESM1_ESM.pdf]

CUL4A$^{DET1\text{-}COP1}$ and OTUD7B Regulate Sox2 Proteostasis and Govern Neural Progenitor Cell Differentiation

Chun-Ping Cui et al

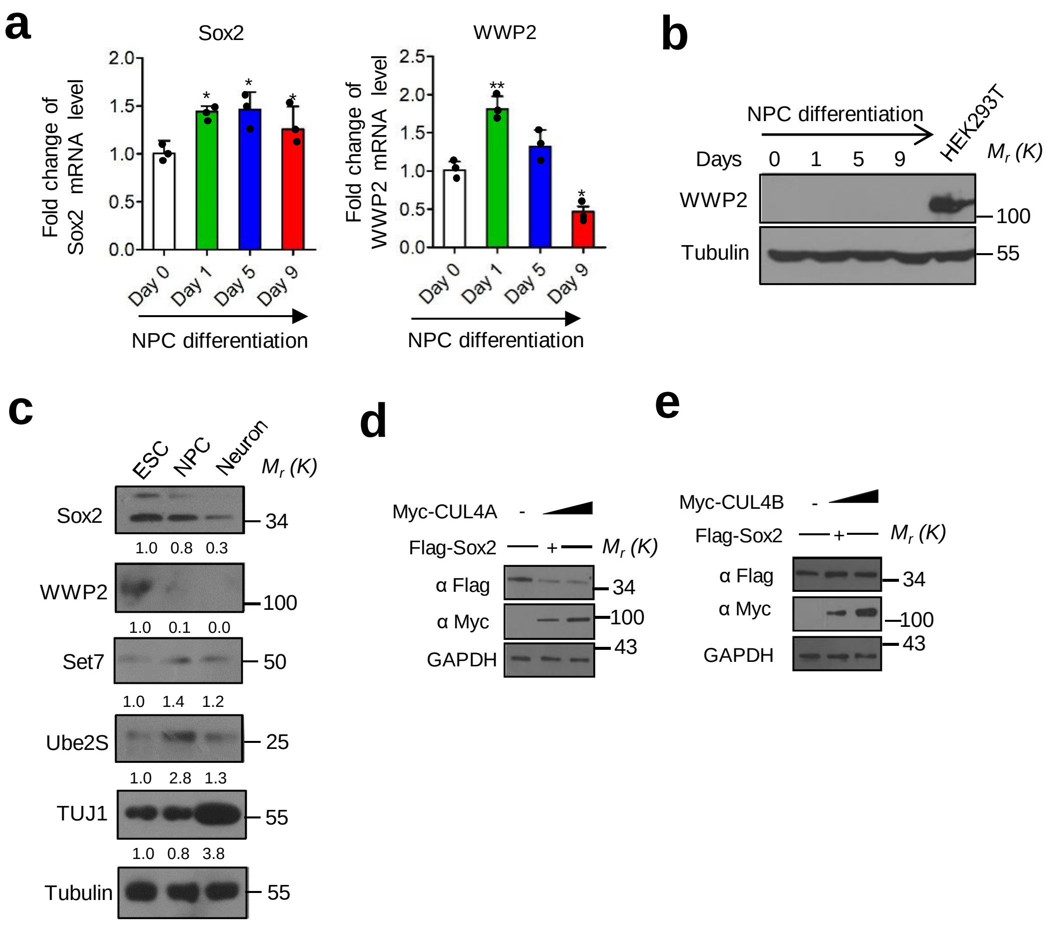

Supplementary Figure 1. CUL4A contributes to Sox2 ubiquitylation during neuronal differentiation. **(a)** mRNA level of Sox2 and WWP2 was measured during NPCs differentiation by Q-PCR. NPCs (neural stem/progenitor cells derived from human PSCs, WA09) were cultured in NIM (neural induction medium) to undergo cellular differentiation for the indicated times. Results are shown as mean ± sd. Each error bar shows the standard deviation of the value from 3 independent experiment. *, P<0.05, **, P<0.01 compared with Day 0, Student's t-test. **(b)** WWP2 protein level was detected by immunoblotting in NPCs and HEK293T (as positive control). **(c)** WWP2, Set7 and Ube2S protein level was detected by immunoblotting in ESC, NPCs and neurons. **(d, e)** Increasing amounts of CUL4A **(d)** or CUL4B **(e)** were transfected into HEK293T cells and ectopic Flag-Sox2 expression was detected. The representative images are shown from 3 independent experiments. Unprocessed original scans of blots are shown in Supplementary Fig. 9.

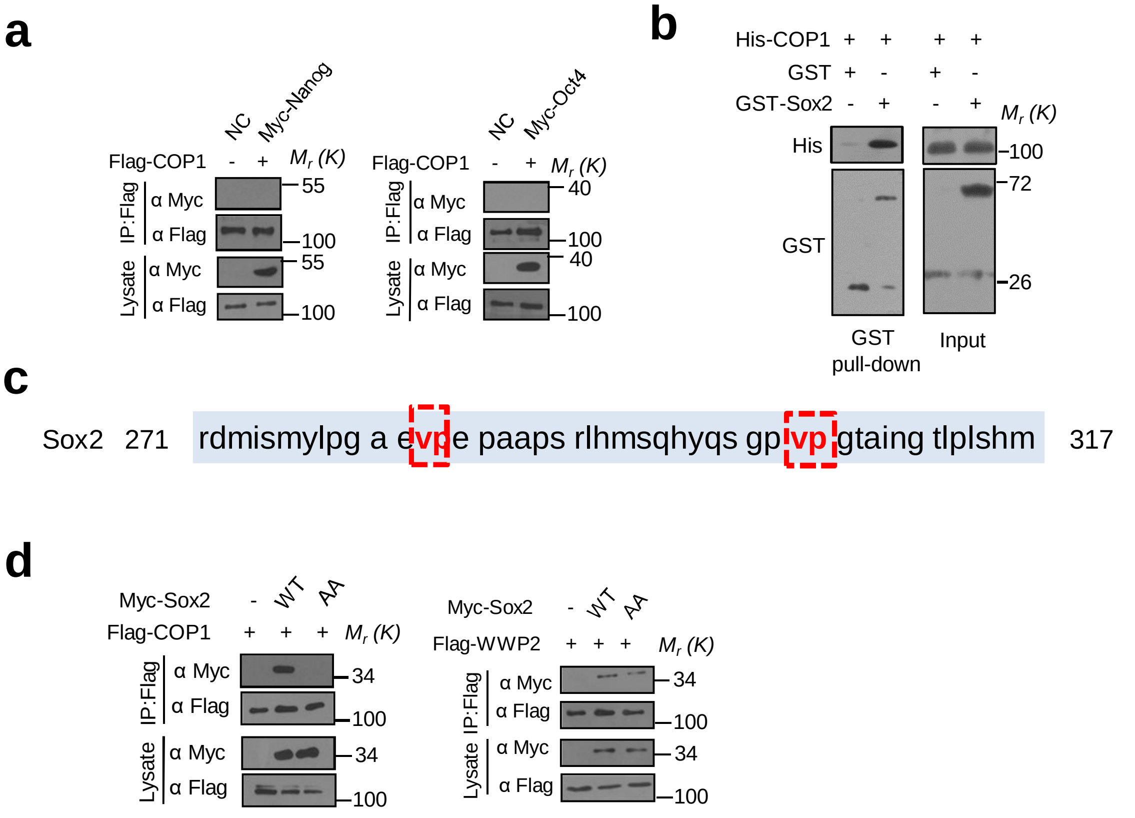

Supplementary Figure 2. The $CUL4A^{DET1\text{-}COP1}$ interacts with Sox2 and regulates its stability.
(a) The lysates of HEK293T transfected with indicated constructs were subjected to immunoprecipitation with anti-Flag antibody. The immunoprecipitates were then blotted. **(b)** The purified His-COP1 were incubated respectively with purified GST or GST-Sox2. The mixtures were subjected to GST pulldown and blotted. **(c)** Diagram of two COP1-recognized VP motif of Sox2. (d) Flag-WWP2 or Flag-COP1 were co-transfected into HEK293T cells with empty vector, Myc-Sox2 WT or Myc-Sox2 AA mutant, respectively. Cell lysates were subjected to immunoprecipitation with anti-Flag antibody and immunoblot. The representative images are shown from 3 independent experiments. Unprocessed original scans of blots are shown in Supplementary Fig. 9.

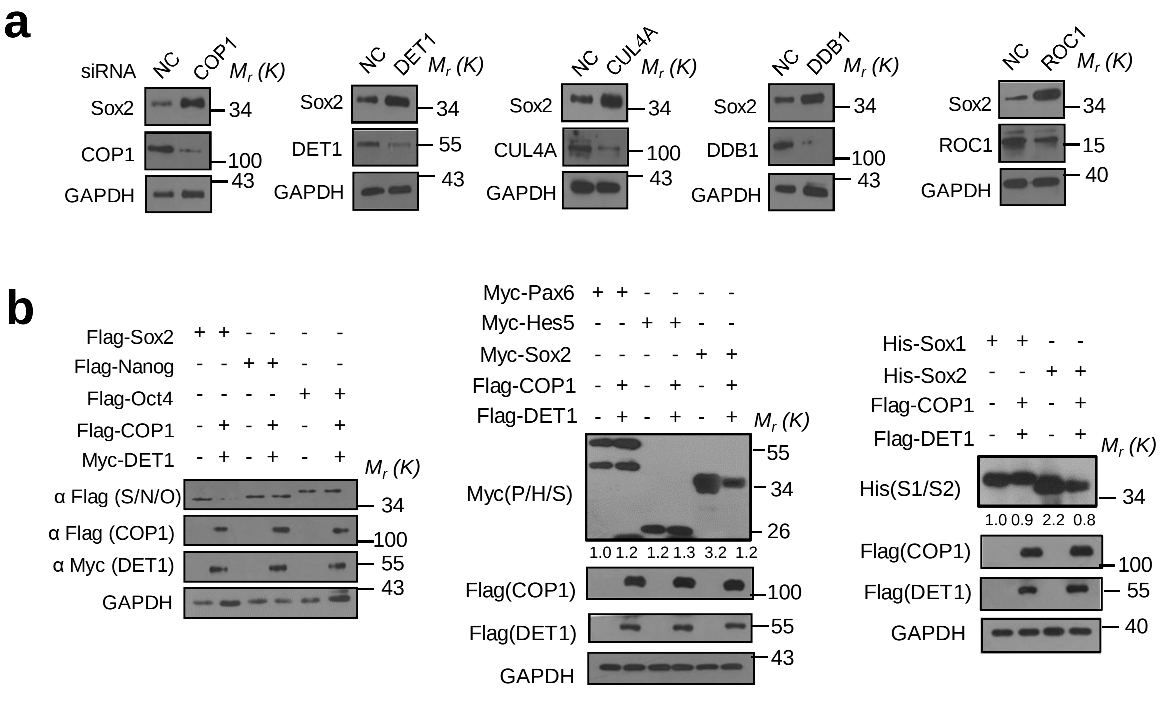

Supplementary Figure 3. COP1 interacts with Sox2 directly.
(a) Cells were transfected with indicated siRNA and western blot was performed to measure expression of Sox2. **(b)** HEK293T cells were transfected with indicated constructs, and the lysates were immunoblotted with anti-Myc, anti-Flag or anti-His. The representative images are shown from 3 independent experiments. Unprocessed original scans of blots are shown in Supplementary Fig. 9.

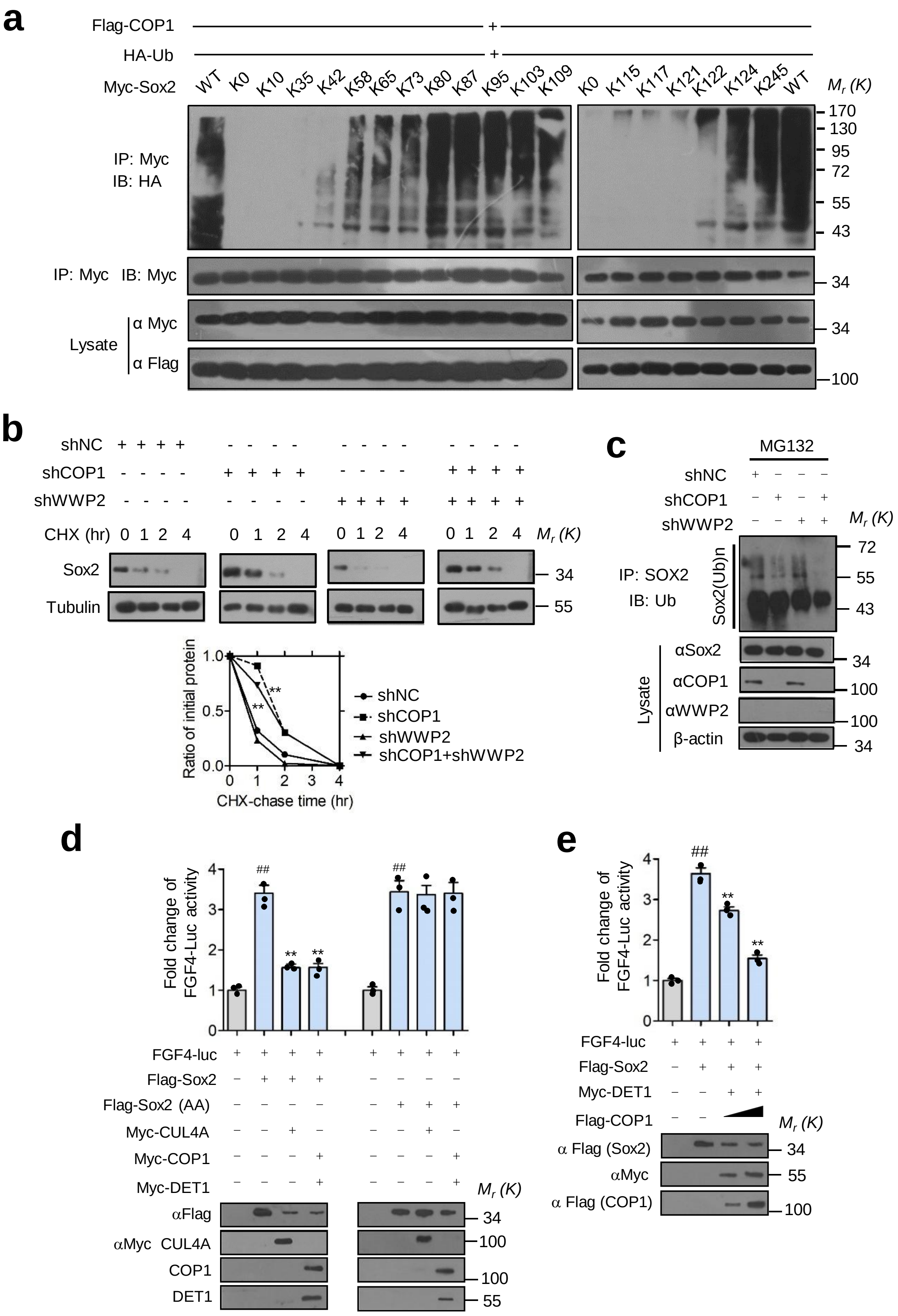

a
Flag-COP1
HA-Ub
Myc-Sox2
WT K0 K10 K35 K42 K58 K65 K73 K80 K87 K95 K103 K109 K0 K115 K117 K121 K122 K124 K245 WT
IP: Myc
IB: HA
IP: Myc IB: Myc
Lysate
α Myc
α Flag
M_r (K)
170
130
95
72
55
43
34
34
100
b
shNC
shCOP1
shWWP2
CHX (hr)
Sox2
Tubulin
34
55
Ratio of initial protein
CHX-chase time (hr)
shNC
shCOP1
shWWP2
shCOP1+shWWP2
c
MG132
shNC
shCOP1
shWWP2
IP: SOX2
IB: Ub
Sox2(Ub)n
72
55
43
Lysate
αSox2
αCOP1
αWWP2
β-actin
34
100
100
34
d
Fold change of FGF4-Luc activity
FGF4-luc
Flag-Sox2
Flag-Sox2 (AA)
Myc-CUL4A
Myc-COP1
Myc-DET1
αFlag
αMyc CUL4A
COP1
DET1
34
100
100
55
e
Fold change of FGF4-Luc activity
FGF4-luc
Flag-Sox2
Myc-DET1
Flag-COP1
α Flag (Sox2)
αMyc
α Flag (COP1)
34
55
100

Supplementary Figure 4. CUL4A$^{DET1\text{-}COP1}$ ubiquitylates Sox2.

(a) HEK293 cells transfected with indicated constructs were treated with MG132 before collection. Myc-Sox2 were immunoprecipated with anti-Myc and immunoblotted with anti-HA. **(b)** NPCs with stable knockdown of COP1, WWP2 or both of them were generated by lentiviral infection. Cell were treated with 10 µg per ml CHX for indicated time. Then Sox2 was immunoblot with anti-Sox2. **(c)** NPCs with stable knockdown of COP1, WWP2 or both of them were generated by lentiviral infection. Cells were treated with MG132 for 8h before collection. Cell lysates were immunoprecitated by anti-Sox2 antibody and then blotted by anti-Ub antibody. **(d, e)** Dual luciferase reporter assay was used to examine the effects of CUL4A$^{DET1\text{-}COP1}$ on the activity of Sox2 in regulating FGF4 transcription. Cells were transfected with indicated constructs. Forty-eight hours after transfection, cell lysates were subjected to dual luciferase reporter assay. The data are normalized to the activity of the cells transfected with empty vectors and presented as the mean ± sd. (##: P<0.01 compared to the cells transfected only with FGF4-luc; **: *P*<0.01 compared to the cells transfected with Flag-Sox2 and FGF4-luc). The expression levels of CUL4A, COP1, DET1 and Sox2 were monitored by western blot with antibodies against Flag and HA, respectively. The representative images are shown from 3 independent experiments. Unprocessed original scans of blots are shown in Supplementary Fig. 9.

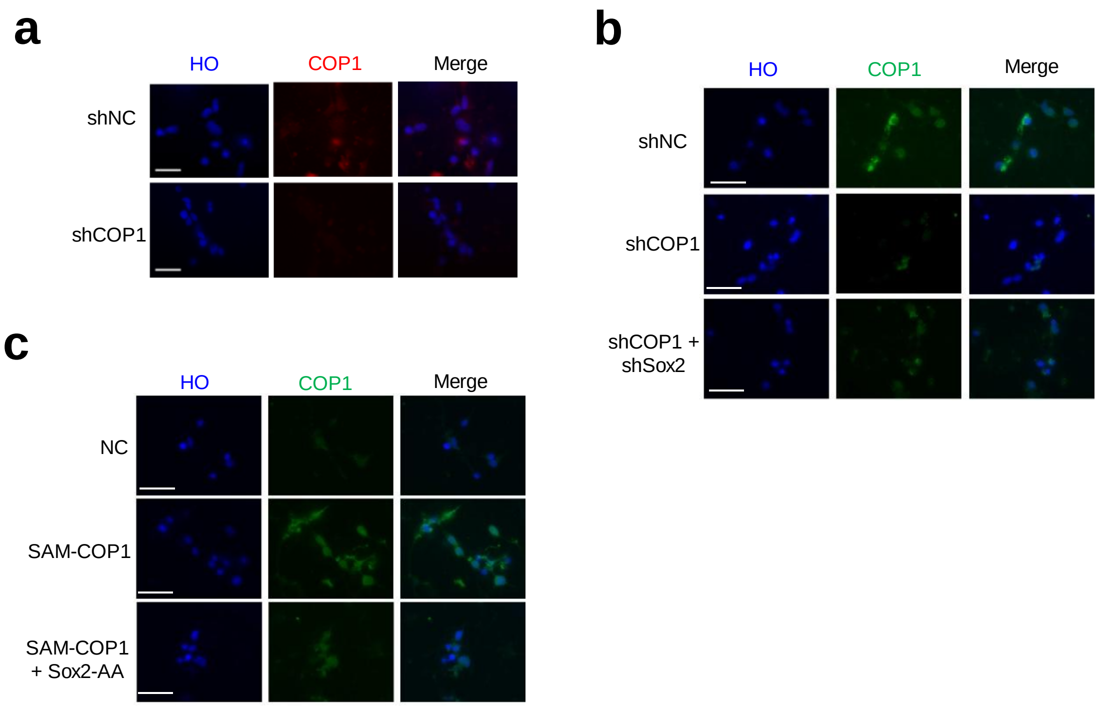

Supplementary Figure 5. COP1 promotes NPCs differentiation in a Sox2-dependent manner. COP1 (red) levels were detected with immunostaining in NPCs with stable COP1 knockdown **(a),** combined knockdown of COP1 and Sox2 **(b),** as well as combined overexpression of COP1 and Sox2-AA **(c).** Nuclei were counterstained with HO (blue). Scale bar, 50 µm. The representative images are shown from 3 independent experiments.

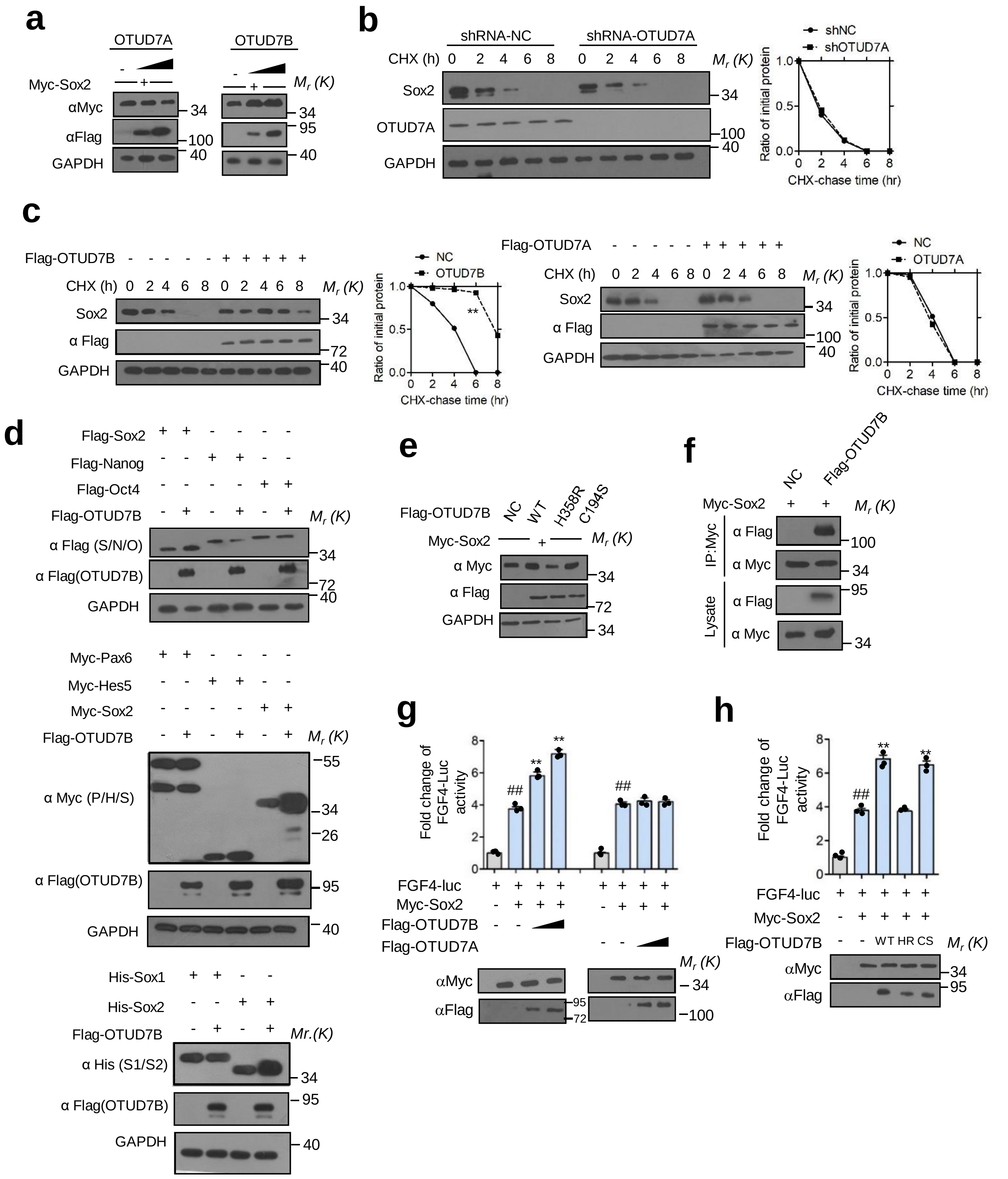

a
OTUD7A
OTUD7B
Myc-Sox2
αMyc
αFlag
GAPDH
M_r (K)
b
shRNA-NC
shRNA-OTUD7A
CHX (h) 0 2 4 6 8 0 2 4 6 8
Sox2
OTUD7A
GAPDH
shNC
shOTUD7A
Ratio of initial protein
CHX-chase time (hr)
c
Flag-OTUD7B
CHX (h) 0 2 4 6 8 0 2 4 6 8
Sox2
α Flag
GAPDH
NC
OTUD7B
**
Ratio of initial protein
CHX-chase time (hr)
Flag-OTUD7A
CHX (h) 0 2 4 6 8 0 2 4 6 8
Sox2
α Flag
GAPDH
NC
OTUD7A
Ratio of initial protein
CHX-chase time (hr)
d
Flag-Sox2
Flag-Nanog
Flag-Oct4
Flag-OTUD7B
α Flag (S/N/O)
α Flag(OTUD7B)
GAPDH
Myc-Pax6
Myc-Hes5
Myc-Sox2
Flag-OTUD7B
α Myc (P/H/S)
α Flag(OTUD7B)
GAPDH
His-Sox1
His-Sox2
Flag-OTUD7B
α His (S1/S2)
α Flag(OTUD7B)
GAPDH
Mr.(K)
e
Flag-OTUD7B
NC WT H358R C194S
Myc-Sox2
α Myc
α Flag
GAPDH
f
NC
Flag-OTUD7B
Myc-Sox2
IP:Myc
Lysate
α Flag
α Myc
g
Fold change of FGF4-Luc activity
##
**
FGF4-luc
Myc-Sox2
Flag-OTUD7B
Flag-OTUD7A
αMyc
αFlag
h
Fold change of FGF4-Luc activity
##
**
FGF4-luc
Myc-Sox2
Flag-OTUD7B
WT HR CS
αMyc
αFlag

Supplementary Figure 6. OTUD7B deubiquitylates and stabilizes Sox2.

(a) The effect of increased OTUD7B or OTUD7A level on Sox2 expression in cells. Cells were transfected with indicated constructs and western blot was performed to measure expression of Flag-Sox2. **(b)** Half-life analysis of Sox2 in cells with OTUD7A knockdown. Cells transfected by indicated shRNA were treated with 10 µg per ml CHX, and collected at the indicated times for western blot. **(c)** Half-life analysis of Sox2 in cells with OTUD7A or OTUD7B overexpression. Cells transfected by indicated constructs were treated with 10 µg/ml CHX, and collected at the indicated times for western blot. Quantification of Sox2 levels relative to tubulin is shown. ∗∗, *P*<0.01, two-way ANOVA test. **(d)** Cells transfected with indicated constructs were lysed and subjected to western blot with anti-Flag , anti-Myc or anti-His antibody. **(e)** Myc-Sox2 was co-transfected into HEK293T cells with Flag-OTUD7B or the mutants, and the cell lysates were immunoprecipated with anti-Myc antibody for detection of the interaction of ectopic Sox2 (or Sox2 mutants) and OTUD7B in cells. **(f)** The interaction of ectopic Sox2 and OTUD7B were detected in HEK293T cells. The lystates of HEK293T cells co-transfected with Myc-Sox2 and Flag-OTUD7B were subjected to immunoprecipitation with anti-Myc antibody and immunoblotted. **(g, h)** Dual luciferase reporter assay was used to examine the effects of OTUD7B on the activity of Sox2 in regulating FGF4 transcription. Cells were transfected with indicated constructs. Forty-eight hours after transfection, cell lysates were subjected to dual luciferase reporter assay. The data are normalized to the activity of the cells transfected with empty vectors and presented as the mean ± S.D. (##: *P*<0.01 compared to the group transfected only with FGF4-luc; **: *P*<0.01 compared to the group transfected with Flag-Sox2 and FGF4-luc). The expression levels of OTUD7B and Sox2 were monitored by westernblot with antibodies against Flag and Myc respectively. The representative images are shown from 3 independent experiments. Unprocessed original scans of blots are shown in Supplementary Fig. 9.

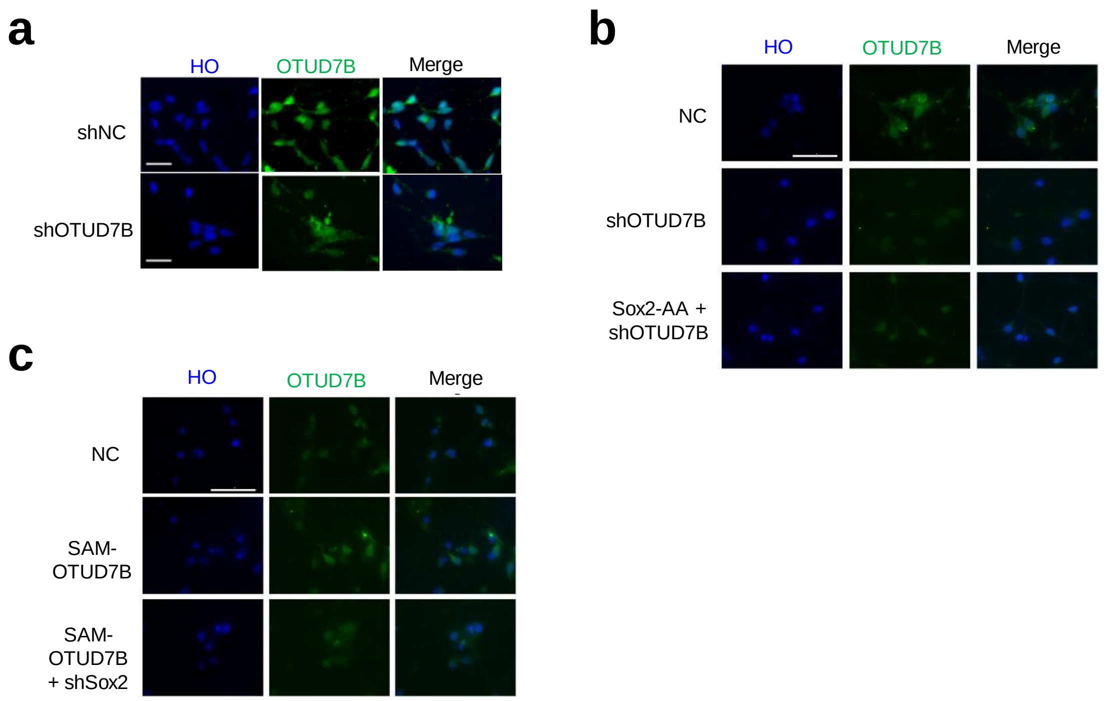

Supplementary Figure 7. OTUD7B maintains NPCs stemness in a Sox2-dependent manner. OTUD7B (green) levels were detected with immunostaining in NPCs with stable OTUD7B knockdown **(a)**, combined OTUD7B knockdown with Sox2-AA overexpression **(b),** as well as combined OTUD7B overexpression with Sox2 knockdown **(c).** Nuclei were counterstained with HO (blue). Scale bar, 50 µm. The representative images are shown from 3 independent experiments.

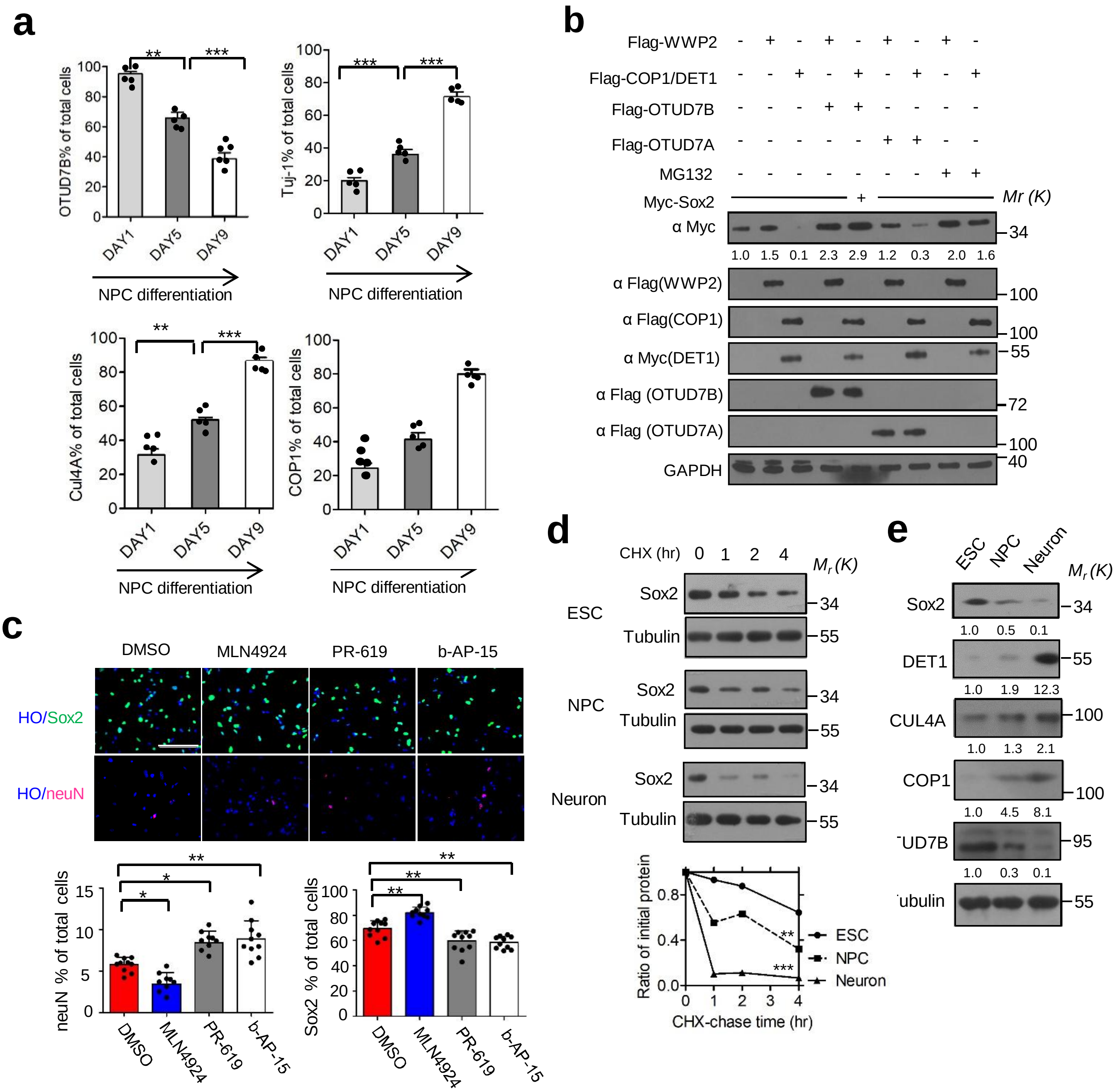

Supplementary Figure 8. COP1 and OTUD7B coordinate to control NPCs differentiation.
(a) Quantified data indicated the fraction of cells expressing Sox2, TUJ1, COP1 or OTUD7B during NPCs differentiation. Data are means ± sd. Each error bar shows the standard deviation of numbers of positive cells in 5 fields of view. **, P<0.01, ***, P<0.001. Student's t-test. **(b)** Cell transfected with indicated constructs were treated with or without MG132 for 8 h. Sox2 level was measured by immunoblotting with anti-myc antibody. **(c)** NPCs were treated with DMSO, MLN4924 (13.3 μM), PR-619 (25 μM) and b-AP-15 (20 μM) for 24h, and then the levels of Sox2 (green) and neuN (cherry) were determined with immunostaining. Nuclei were counterstained with HO (blue). Scale bar, 50 μm. Each error bar shows the standard deviation of numbers of positive cells in 10 fields of view. *, *P*<0.05, **, *P*<0.01. Student's t-test. **(d)** ESCs, NPCs and neurons were treated with 10 μg per ml CHX, and collected at the indicated time for western blot. Quantification of Sox2 levels relative to tubulin is shown. **: *P*<0.01, ***: *P*<0.001, two-way ANOVA test. **(e)** Sox2, DET1, CUL4A, COP1 and OTUD7B protein level was detected by immunoblotting in ESC, NPCs and neurons. The representative images are shown from 3 independent experiments. Unprocessed original scans of blots are shown in Supplementary Fig. 9.

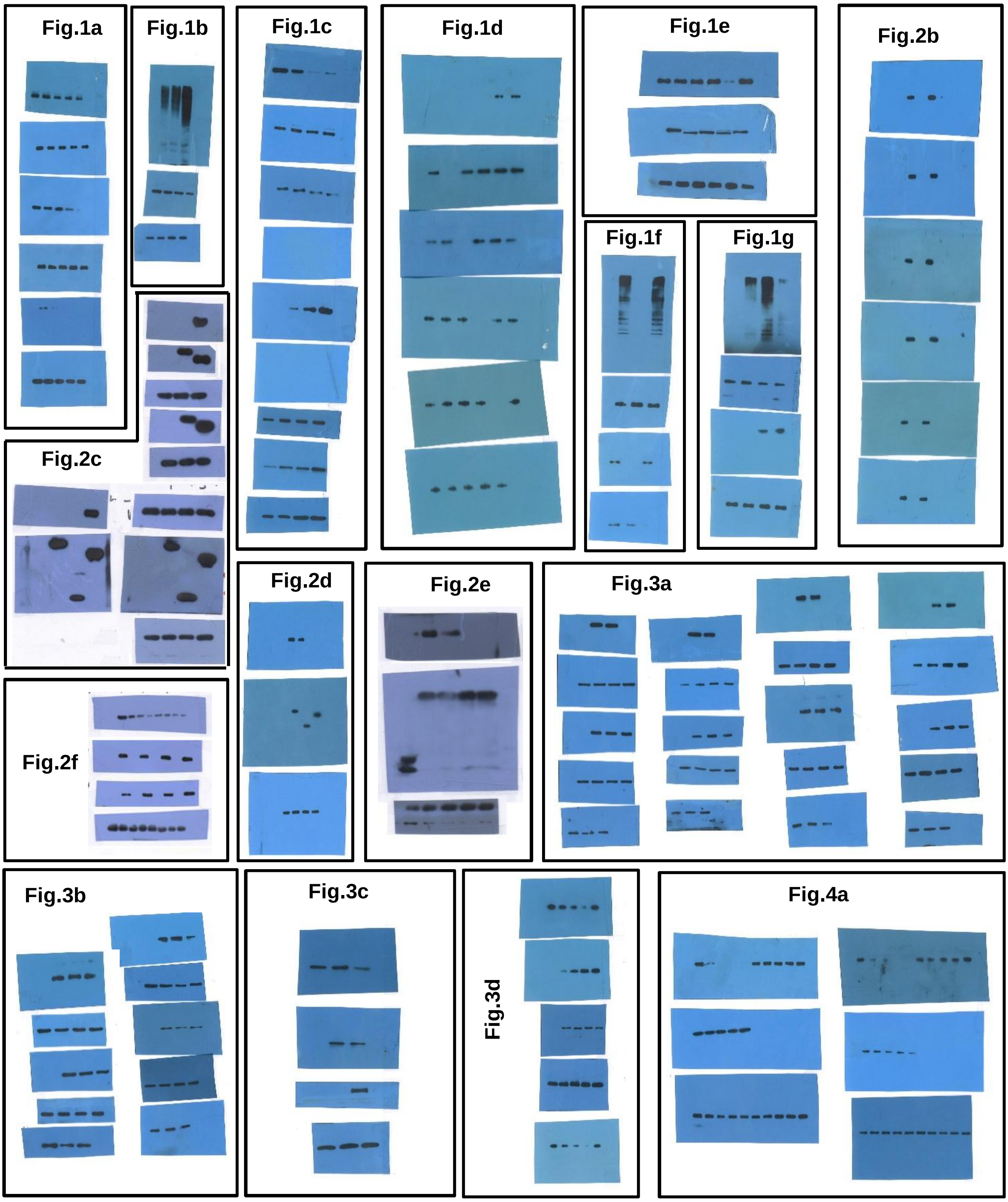

Supplementary Figure 9. Uncropped scans of Western blots.

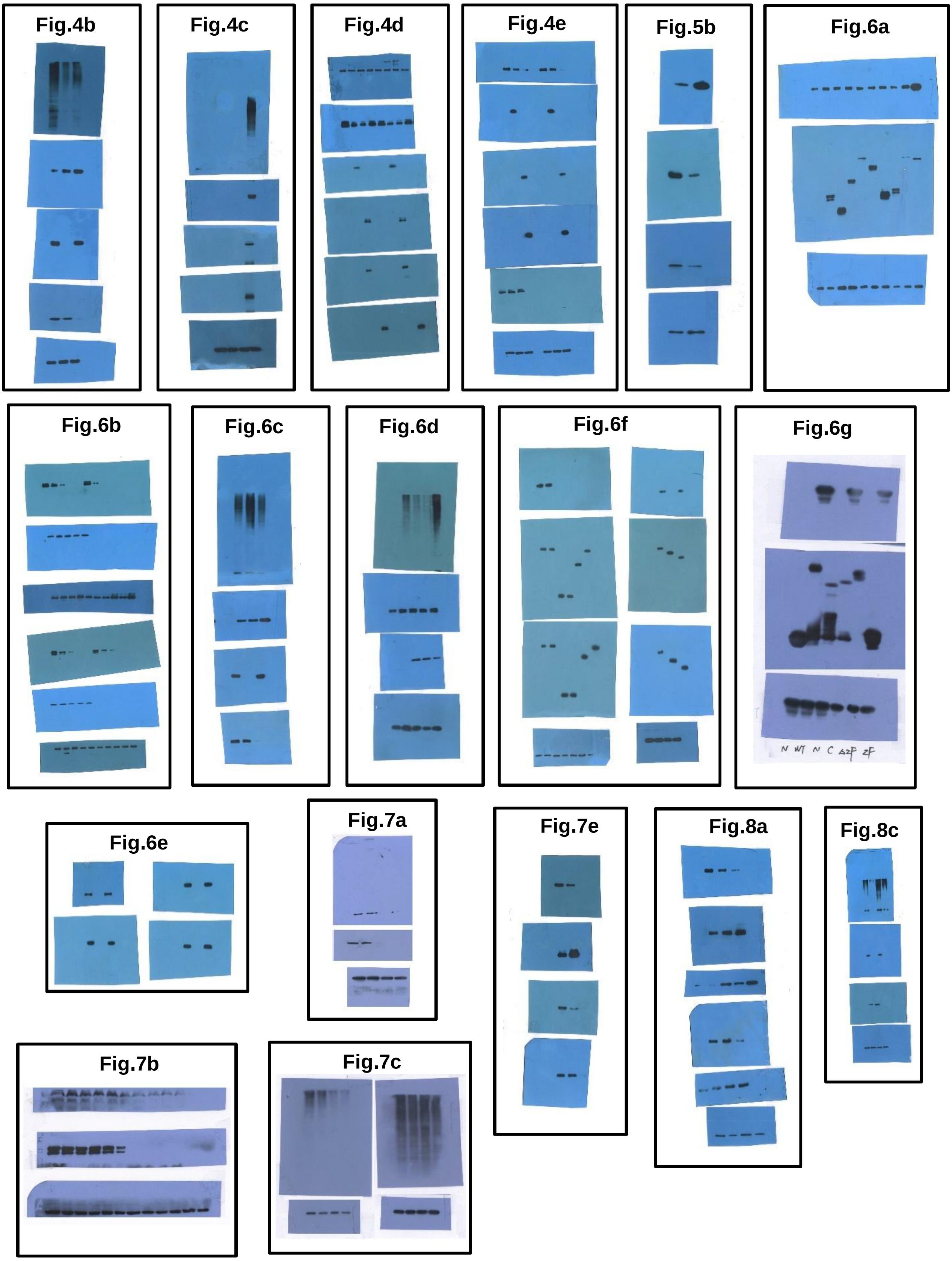

Supplementary Figure 9. Uncropped scans of Western Blots (Continuation 1).

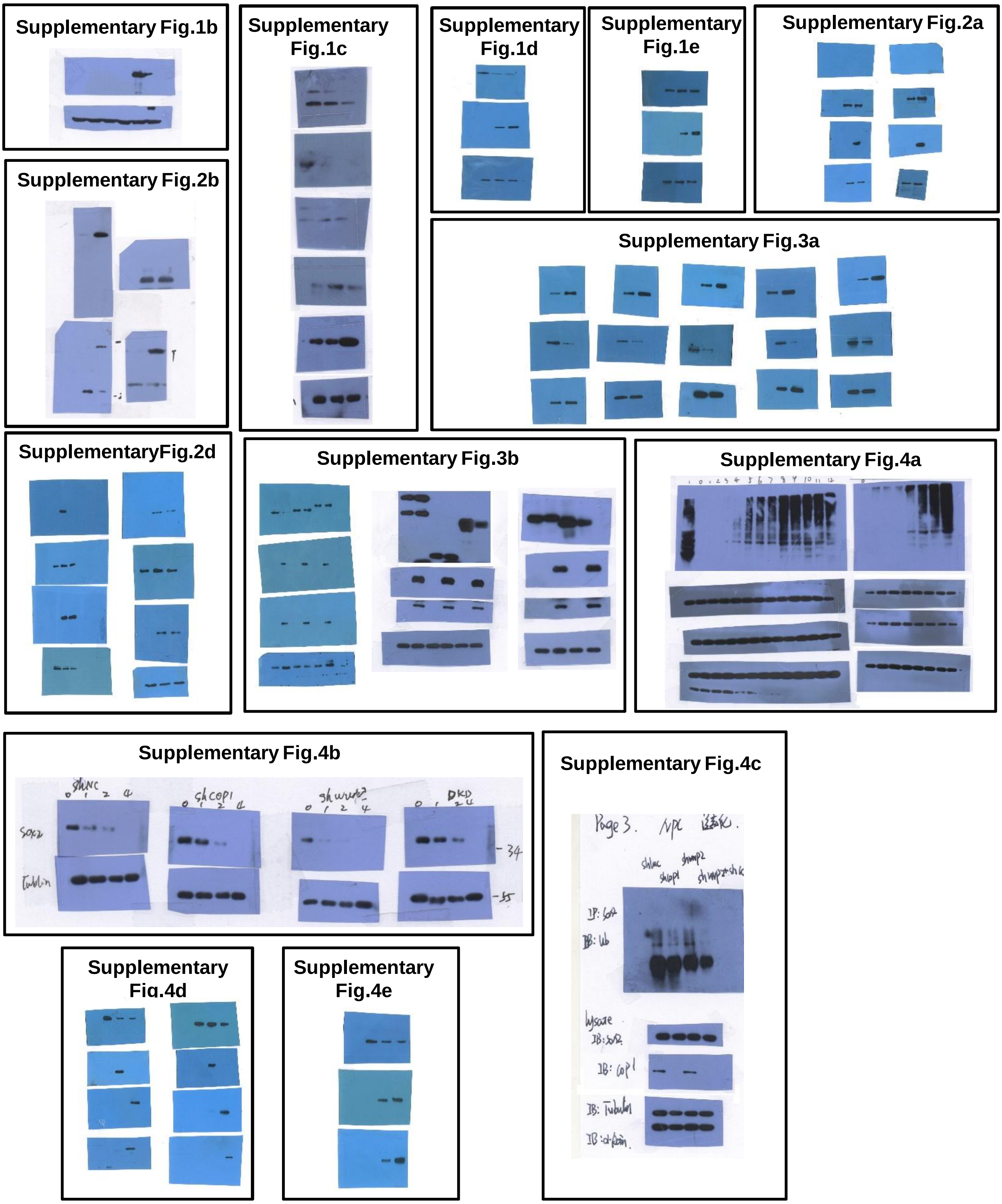

Supplementary Figure 9. Uncropped scans of Western Blots (Continuation 2).

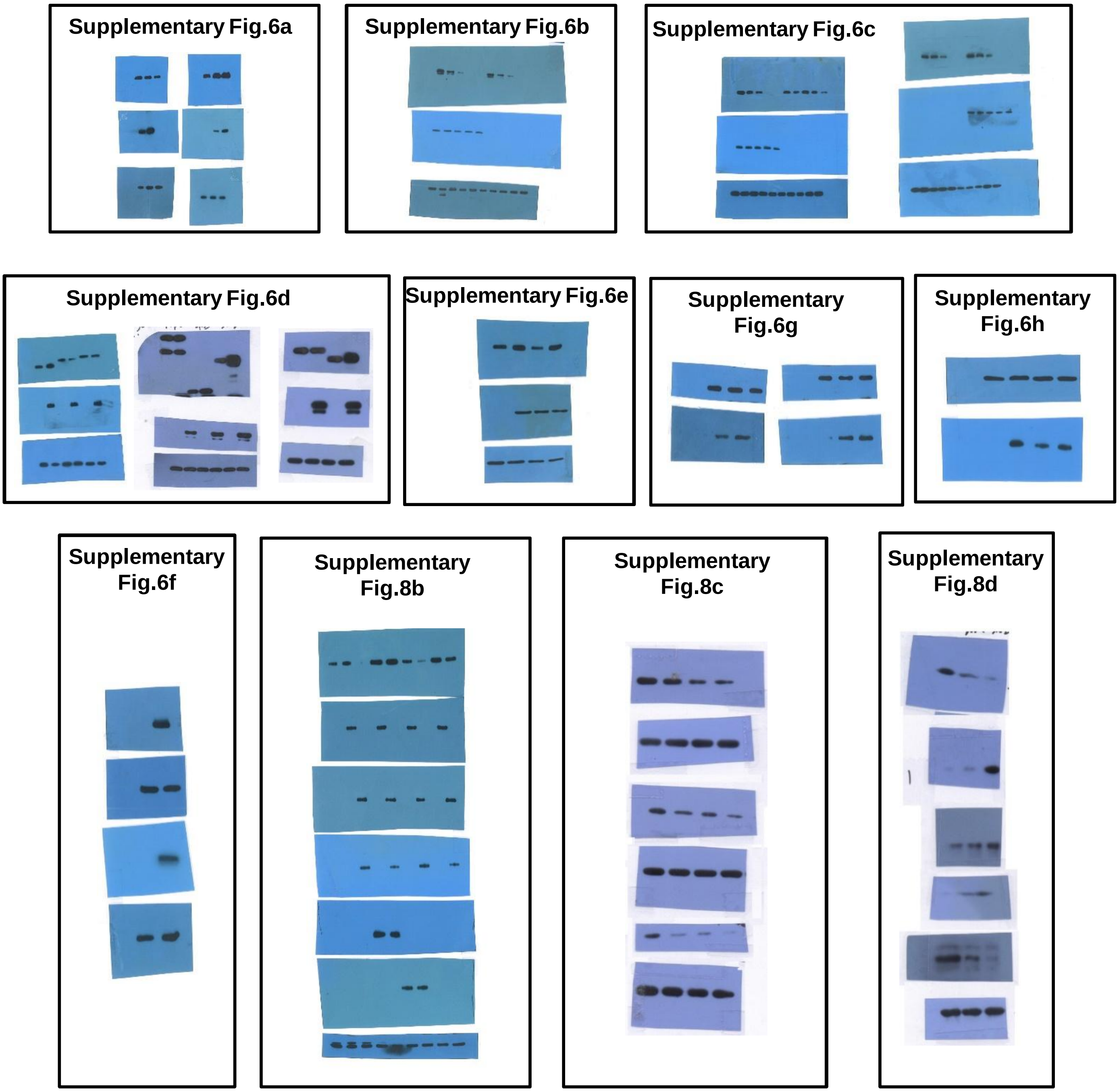

Supplementary Figure 9. Uncropped scans of Western lots (Continuation 3).
