# Supplementary material for: Dynamic ubiquitylation of Sox2 regulates proteostasis and governs neural progenitor cell differentiation
Source: Nat Commun. 2018 Nov 7;9:4648. doi: 10.1038/s41467-018-07025-z (PMC6220269; doi:10.1038/s41467-018-07025-z)
Supplement: Supplementary file 3 — Description of Additional Supplementary Files [file 41467_2018_7025_MOESM3_ESM.docx]

**Description of Additional Supplementary Files**

File Name: Supplementary Data 1

Description: Predicted Interaction of Sox2 and E3 ligases in UbiBrowse. Eighty four predicted E3 ligases in UbiBrowser are presented. These E3s were classified into six different families, Ring, HECT, U-Box, Complex-DWD, Complex-CDC20, and Single. PFAM means the likelihood ratio of most enriched domain pair; GO means the likelihood ratio of enriched GO pair; NET means the likelihood ratio of network loops (including  three-interaction loops and four-interaction loops); MOTIF means the likelihood of ratio of E3 recognizing motif; SCORE means confidence score of brief information.

File Name: Supplementary Data 2

Description: List of primers used for plasmids construction. The primers used for plasmid construction in our experiments are presented.
